# Biomimetic strain-stiffening in fully synthetic dynamic-covalent hydrogel networks†

**DOI:** 10.1039/d3sc00011g

**Published:** 2023-04-13

**Authors:** Rachel C. Ollier, Yuanhui Xiang, Adriana M. Yacovelli, Matthew J. Webber

**Affiliations:** a Department of Chemical & Biomolecular Engineering, University of Notre Dame Notre Dame IN 46556 USA mwebber@nd.edu

## Abstract

Mechanoresponsiveness is a ubiquitous feature of soft materials in nature; biological tissues exhibit both strain-stiffening and self-healing in order to prevent and repair deformation-induced damage. These features remain challenging to replicate in synthetic and flexible polymeric materials. In recreating both the mechanical and structural features of soft biological tissues, hydrogels have been often explored for a number of biological and biomedical applications. However, synthetic polymeric hydrogels rarely replicate the mechanoresponsive character of natural biological materials, failing to match both strain-stiffening and self-healing functionality. Here, strain-stiffening behavior is realized in fully synthetic ideal network hydrogels prepared from flexible 4-arm polyethylene glycol macromers *via* dynamic-covalent boronate ester crosslinks. Shear rheology reveals the strain-stiffening response in these networks as a function of polymer concentration, pH, and temperature. Across all three of these variables, hydrogels of lower stiffness exhibit higher degrees of stiffening, as quantified by the stiffening index. The reversibility and self-healing nature of this strain-stiffening response is also evident upon strain-cycling. The mechanism underlying this unusual stiffening response is attributed to a combination of entropic and enthalpic elasticity in these crosslink-dominant networks, contrasting with natural biopolymers that primarily strain-stiffen due to a strain-induced reduction in conformational entropy of entangled fibrillar structures. This work thus offers key insights into crosslink-driven strain-stiffening in dynamic-covalent phenylboronic acid–diol hydrogels as a function of experimental and environmental parameters. Moreover, the biomimetic mechano- and chemoresponsive nature of this simple ideal-network hydrogel offers a promising platform for future applications.

## Abstract 2

Strain-stiffening is observed and characterized in flexible PEG hydrogel networks crosslinked *via* dynamic-covalent boronate ester bonds, revealing an uncommon synthetic mimic of a mechanoresponse found in natural biopolymer networks.

## Graphical abstract

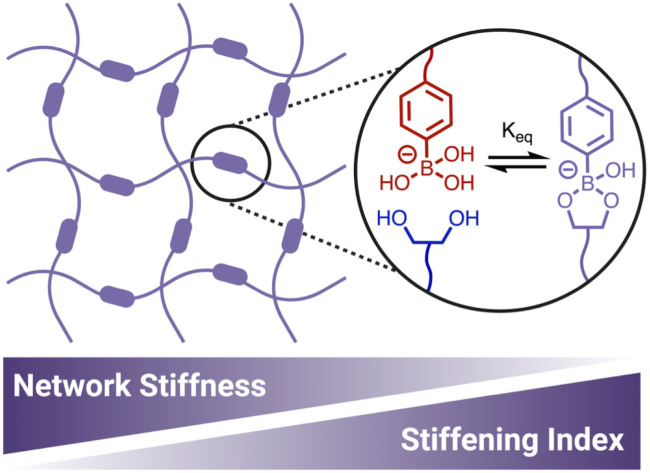

## Introduction

**Fig. 1:**
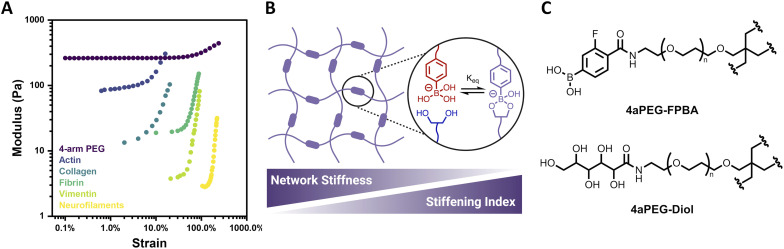
Structure and strain-dependent stiffness of 4-arm PEG hydrogels. (A) Strain-stiffening of 4-arm PEG hydrogel (2.5 wt%, pH 6.5, 25 °C) relative to that of various biopolymer networks. Biopolymer data reproduced from Storm *et al.* (ref. 1) (B) ideal network hydrogel with dynamic-covalent PBA–diol crosslinks, reported here to have network stiffness and stiffening index that are inversely related. (C) Structures of 4-arm PEG FPBA (4aPEG-FPBA) and 4-arm PEG diol (4aPEG-diol) macromers.

Biological tissues often exhibit mechanoresponsive character, with mechanical stimuli initiating structural changes that prevent large deformations and thereby protect and preserve biological function.^1^ Such responses commonly include the capacity of biological materials to both strain-stiffen and self-heal. Strain-stiffening, a process by which materials become stiffer under applied deformation, is ubiquitous among fibrous proteins such as actin, collagen, fibrin, and vimentin (Fig. 1A).^2,3^ Strain-stiffening is a mechanical response characterized by a deviation from linear viscoelasticity as the applied strain increases. For natural materials, this strain-dependent deviation is typically attributed to fibrillar structures that engage in bundling and physical entanglements.^4,5^ Accordingly, natural protein materials act as semiflexible networks that provide necessary mechanical properties, including stiffening in response to strain, and thereby underlie functional biological tissues as well as processes such as cell differentiation, motility, and communication.^6–11^

Self-healing is another mechanical response commonly found in biological tissues; deformation-induced damage is autonomously repaired, restoring the mechanical properties of the original tissue.^12^ In biological tissues, however, self-healing repair typically occurs on a relatively long timescale, encompassing distinct phases of inflammation, proliferation, and remodeling.^13^ Depending on the extent of damage, the repair process can take months or even years. Synthetic materials, on the other hand, typically lack the complexity of biological tissues and thus undergo rapid repair with indistinguishable healing phases.^14^ Self-healing polymer and composite materials often leverage intrinsic healing through the selection of dynamic functional groups or gelation mechanisms that enable self-healing following an applied stimulus.^13^ This can include equilibrium-governed dynamic and/or supramolecular modes of network crosslinking.^15–17^ More active designs include capsule and vascular-based systems, wherein active healing agents are stored in compartments of the bulk material and released upon damage to initiate repair.^18–20^

Hydrogels have been widely explored as candidates for applications in tissue engineering, drug delivery, and wound dressings due to their biocompatibility as well as their mechanical and structural similarities to soft biological tissues.^21–23^ One route to prepare synthetic hydrogels is by dynamic-covalent crosslinking, leveraging inherent dynamic exchange of boronate esters, disulfides, imines, and other equilibrium-governed dynamic-covalent bonds.^7,15,24,25^ However, dynamic-covalent hydrogels, and particularly those prepared from synthetic polymers, rarely replicate the strain-stiffening mechanoresponse of natural materials. Incorporating both strain-stiffening and self-healing functionalities in fully synthetic hydrogels has therefore been a significant challenge.

Here, the strain-stiffening behavior of synthetic ideal-network hydrogels is explored using 4-arm polyethylene glycol (PEG) macromer networks with dynamic-covalent boronate ester crosslinks (Fig. 1B). The dynamic-covalent bond between a phenylboronic acid (PBA) and a *cis*-1,2 or *cis*-1,3 diol has been widely explored in preparing glucose-responsive materials due to its susceptibility to competition from free glucose.^26–32^ The PBA–diol interaction is also sensitive to environmental parameters such as pH or temperature, with bonds formed more readily in pH conditions at or above the p*K*_a_ of the specific PBA motif and bond dynamics being accelerated with increased temperature. This has led to the preparation of biomimetic PBA–diol crosslinked hydrogels responsive to a variety of stimuli.^33,34^ However, the strain-stiffening behavior of this class of materials has not yet been investigated. Herein, strain-stiffening in PEG-based boronate ester hydrogels is thoroughly explored as a function of different hydrogel parameters, including concentration, pH, and temperature. The reversibility of the strain-stiffening response in these hydrogels is also demonstrated over multiple cycles of strain. Accordingly, the biomimetic strain-stiffening and self-healing character of this simple synthetic system introduces added mechanical functionality for various biologically-relevant applications.

## Results & discussion

### Hydrogel design

Four-arm polyethylene glycol (4aPEG, 10 kDa) macromers were end-modified with 4-carboxy-3-fluorophenylboronic acid (FPBA) *via* amide formation on a 4aPEG-NH_2_ precursor to prepare the PBA-modified macromer (4aPEG-FPBA, Fig. 1C), as previously described.^26^ This FPBA motif was chosen for its physiologically relevant p*K*_a_ value of ∼7.2–7.3,^26,35^ ensuring the boronate would be in a diol-binding conformation over a roughly neutral pH range. A diol-modified macromer (4aPEG-diol, Fig. 1C) was similarly prepared by reacting 4aPEG-NH_2_ with glucono-δ-lactone, as previously reported.^29^ This hydrogel design was intended to facilitate formation of an ideal network, and benefits from the use of 4aPEG macromer precursors to yield a defined length of elastically active network strands. Prior work demonstrated canonical Maxwell behavior for these materials, evident in terminal relaxation behavior and concentration-independent *G*′/*G*′′ crossover.^26^ The overlap concentration (c*) of 4aPEG macromers in water, calculated previously to be ∼11.5 wt%, furthermore supports limited entanglements over the concentration regime of 2–5 wt% to be explored here.^26,36,37^ Accordingly, an ideal or “ideal-like” assumption is reasonable for the primary hydrogel network under study in this work. When the 4aPEG-FPBA and 4aPEG-diol macromers were initially mixed 1 : 1 at an overall concentration of 2.5 wt% and assessed by dynamic oscillatory rheology, stiffening was observed, apparent from an increase in the storage modulus (*G*′) as the amplitude of strain was increased (Fig. 1A). Such a response is highly uncommon in fully synthetic hydrogels. Accordingly, this early observation led to a multi-parametric study to understand and classify the nature of this strain-stiffening response.

### Concentration-dependent strain-stiffening

**Fig. 2:**
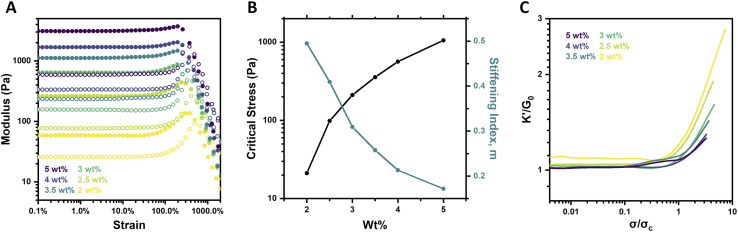
Concentration-dependent strain-stiffening behavior of 4-arm PEG gels (pH 6.5, 25 °C). (A) Storage modulus (*G*′, filled circles) and loss modulus (*G*′′, empty circles) as a function of strain for 2–5 wt% gels. (B) Critical stress (*σ*_c_) and stiffening index (*m*) as a function of polymer concentration in hydrogels. (C) Differential modulus (*K*′) normalized by plateau modulus (*G*_0_) as a function of stress (*σ*) normalized by critical stress (*σ*_c_) for 2–5 wt% gels. Stiffening index (*m*) is calculated as the slope of this plot in the stiffening regime.

Altering the concentration of biological materials is known to impact their strain-stiffening response, with elevated concentrations increasing the prevalence of physical crosslinks and fibrillar bundles that underlie strain-stiffening.^5,38^ PEG macromer concentration was thus explored for its impact on strain-stiffening for the system reported here, characterizing 4aPEG hydrogels crosslinked by FPBA–diol interactions prepared over a range of 2–5 wt% at fixed pH (6.5) and temperature (25 °C). Both strain sweeps and frequency sweeps were performed, alongside strain-stiffening analysis as detailed below. The strain sweeps revealed an increase in hydrogel stiffness with increasing concentration; such an effect is predictable given the higher crosslink density of these networks at elevated concentrations (Fig. 2A), and aligns with prior work showing concentration-dependent *G*′ increases in these materials.^26^

Interestingly, when the strain-stiffening response was further investigated, these data revealed that the magnitude of the stiffening response decreased with increasing macromer concentration. For example, the 2 wt% gel stiffened from an initial plateau modulus (*G*_0_) of 59 Pa to a maximum *G*′ of 137 Pa, a ∼130% increase, while the 5 wt% gel stiffened from *G*_0_ of 3.1 kPa to a maximum value of 3.7 kPa for only a ∼20% increase in stiffness (Fig. 2A). These trends were further visualized in plots of the critical stress (*σ*_c_) and the stiffening index (*m*) as a function of concentration (Fig. 2B). The critical stress increased nearly 2 orders of magnitude, from 21.2 Pa to 1055.9 Pa, while the stiffening index decreased from 0.49 to 0.17 as concentration was increased from 2 wt% to 5 wt%. Further analysis is possible by calculating the differential modulus (*K*′) defined as the derivative of shear stress with respect to shear strain, ∂*σ*/∂*γ*. The dramatic difference in the intensity of the stiffening response was evident from plotting *K*′/*G*_0_ as a function of *σ*/*σ*_c_ for each gel concentration (Fig. 2C). Both the intensity and extent of the stiffening response, respectively measured by the slope (quantified as the stiffening index, *m*) and maximum value of *K*′/*G*_0_, increased at lower macromer concentrations.

In order to verify that the strain-stiffening response was not specific to the 4aPEG-FPBA chemistry, networks prepared from an alternate PBA-bearing macromer, 4aPEG-PyPBA (Fig. S1†), and 4aPEG-diol gels were also prepared and characterized at different concentrations (Fig. S2†). Networks prepared from this alternate boronate ester dynamic-covalent bonding likewise demonstrated a concentration-dependent increase in *G*_0_ in the low-strain regime, with a stiffening response evident by an increase in *G*′ at higher strain. Additionally, due to the dynamic nature of the crosslinks in these networks, their mechanical properties are generally independent of the method of preparation. For example, a gel originally prepared at 2.5 wt% that was lyophilized and rehydrated to 5 wt% shows the same strain-stiffening response as a freshly prepared 5 wt% gel (Fig. S3†).

### pH-dependent strain-stiffening

**Fig. 3:**
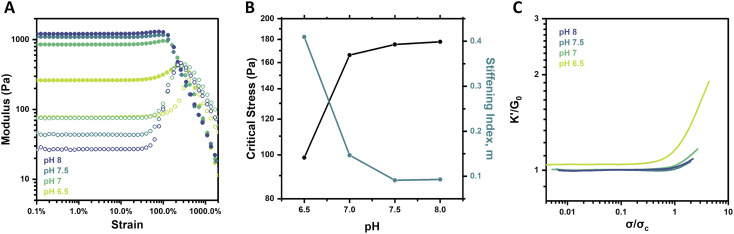
pH-dependent strain-stiffening behavior of 4-arm PEG gels (2.5 wt%, 25 °C). (A) Storage modulus (*G*′, filled circles) and loss modulus (*G*′′, empty circles) as a function of strain for pH 6.5–8 gels. (B) Critical stress (*σ*_c_) and stiffening index (*m*) of gels as a function of pH. (C) Normalized differential modulus (*K*′/*G*_0_) as a function of normalized stress (*σ*/*σ*_c_) for pH 6.5–8 gels.

Though pH is not a common stimulus underlying the strain-stiffening response of natural biological systems, it is a specifically relevant stimulus in the context of PBA–diol bonding. Notably, the formation and stability of boronate ester crosslinks are sensitive to pH, which alters the effective crosslink density of the network.^29^ Below the p*K*_a_ of the specific PBA motif, approximated to be ∼7.2–7.3 for the FPBA motif used here,^26,35^ the neutral boronic acid does not form stable complexes with diols. At or above its p*K*_a_, the negatively charged tetrahedral boronate will readily complex with diols and form stable crosslinks.^39^ The strain-stiffening behavior of these hydrogels was therefore investigated as a function of pH, with hydrogels prepared at pH 6.5, 7, 7.5, and 8 at a constant concentration (2.5 wt%) and temperature (25 °C). Strain sweeps on these gels (Fig. 3A) revealed an increase in hydrogel stiffness with increasing pH. This result corresponds to the increasing fraction of boronate groups available to form stable complexes with diols as pH is increased. Frequency sweeps also showed an increase in *G*′ as well as a reduction in the *G*′/*G*′′ crossover frequency (*ω*_c_) from 12.6 rad s^−1^ at pH 6.5 to 0.6 rad s^−1^ at pH 8 (Fig. S4†), pointing to less dynamic network crosslinks as pH is increased, in alignment with previous reports of PBA–diol networks.^29^

Interestingly, from the strain sweeps, the weakest gel prepared at pH 6.5 showed the most pronounced stiffening, with *G*′ increasing from 262 to 442 Pa (∼69%), while *G*′ of the pH 8 gel only increased from 1212 to 1304 Pa (∼8%) (Fig. 3A). This phenomenon was also reflected in a plot of the critical stress (*σ*_c_) and the stiffening index (*m*) as a function of pH (Fig. 3B). This analysis points to a limited increase in critical stress when gel pH is increased beyond 7, as well as the pronounced decrease in the stiffening index between pH 6.5 and 7 but limited decrease beyond this point. These findings for limited pH-responsive changes above pH 7 align with the expected p*K*_a_ of the FPBA motif used here. This condition is further supported by studies on networks prepared from the alternate PyPBA chemistry (Fig. S5†), which has a p*K*_a_ of ∼4.4.^26^ For this network, both *G*_0_ and the ensuing stiffening behavior at higher strain were relatively pH-independent over the range of 6.5–7.5, all in excess of the p*K*_a_ for this crosslinking chemistry.

When plotting *K*′/*G*_0_ as a function of *σ*/*σ*_c_ for the FPBA hydrogels at each pH, a similar trend was revealed (Fig. 3C). Both the intensity and extent of the stiffening response for the gel at pH 6.5 were much higher than for the gels at pH 7–8. These findings are attributed to the change in effective crosslink density in the network. Because stable PBA–diol complexes form at or above the p*K*_a_ of the PBA, modulating gel pH dictates the extent and duration of crosslinking in the network. Due to the logarithmic nature of pH and p*K*_a_, the impact of pH on crosslink density is diminished as pH is elevated relative to p*K*_a_. Boronate esters have slightly lower p*K*_a_ than their boronic acid counterparts. Accordingly, PBA–diol complexation drives equilibrium to the tetrahedral boronate and promotes the formation of more stable complexes.^39^ This may account for the ability of the macromers to gel, albeit weakly, at pH 6.5. Generally, the relationship between pH-induced changes in gel mechanical properties and strain-stiffening agrees with the concentration-dependent results, as in both cases gels with lower crosslink density stiffen more acutely. Further exploration of hydrogel networks prepared from macromers of different molecular weights, thereby altering the crosslink density of networks prepared at equivalent polymer concentrations, may yield additional insight for the importance of crosslink density on strain-stiffening.

### Interplay of concentration and pH on strain-stiffening

**Fig. 4:**
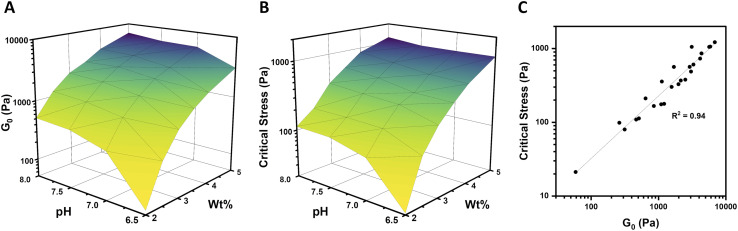
pH- and concentration-dependent mechanical properties of 4-arm PEG hydrogels. (A) Plateau modulus (*G*_0_) as a function of pH (6.5–8) and gel concentration (2–5 wt%). (B) Critical stress (*σ*_c_) as a function of pH and gel concentration. (C) Critical stress as a function of plateau modulus for gels prepared at each combination of pH and concentration.

In order to fully explore the impact of pH and concentration on hydrogel mechanical properties, gels with concentrations over a range of 2–5 wt% were prepared at pH of 6.5, 7, 7.5, and 8, and rheological characterization was performed as before on each of these 24 unique gels. Both the low-strain plateau modulus (*G*_0_) and the critical stress (*σ*_c_) were plotted as a function of both pH and concentration (Fig. 4A and B). The low-strain modulus of each gel increased as both pH and concentration increased, as already rationalized above. Interestingly, critical stress behaved similarly, increasing with both pH and concentration. These plots are useful in understanding the relative changes in mechanical properties due to pH and macromer concentration. Across all pH values, increasing concentration increased *G*_0_ and critical stress by a minimum of one order of magnitude. This increase was most dramatic, spanning nearly 2 orders of magnitude, at pH 6.5. The concentration-dependent changes in both *G*_0_ and critical stress were minimized at higher pH, with gels prepared at pH 7.5 and 8 behaving comparably. Similarly, at elevated gel concentrations, the impact of pH was diminished. At 2 wt%, gels spanning the pH range of 6.5–8 varied by approximately an order of magnitude in both *G*_0_ and critical stress. In contrast, *G*_0_ for 5 wt% gels spanning the pH range of 6.5–8 merely doubled from 3.1 to 6.9 kPa, while the critical stress of these gels increased minimally from 1.1 kPa to 1.2 kPa at pH 6.5 and 8, respectively. When critical stress was plotted as a function of plateau modulus *G*_0_ (Fig. 4C), there was a clear correlation, wherein critical stress increased as 
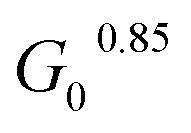
 (*R*^2^ of 0.94). From these data, varying macromer concentration appears to be the most effective means to modulate mechanical properties. This is advantageous for biological applications, which can rarely deviate from physiological pH yet have substantial freedom to alter polymer content within this low concentration regime.

In addition to the trends in dynamics resulting from pH, as previously described, the dynamic properties of these gels also showed some dependence on concentration. Frequency sweeps performed on 2–5 wt% gels prepared at pH 6.5, 7, 7.5, and 8 (Fig. S6–S9†) revealed concentration-dependent crossover frequency that varied with pH. At pH 6.5, *ω*_c_ decreased from 19.9 rad s^−1^ for a 2 wt% gel to 8.6 rad s^−1^ for a 5 wt% gel. This concentration-dependent change in dynamics at pH levels below the p*K*_a_ of the FPBA motif speaks to a concentration-mediated shift in the binding equilibrium of the dynamic-covalent bond. At pH 7.5, however, the crossover frequency was independent of gel concentration at 1.6 rad s^−1^ for all gels. This finding is consistent with previous reports for concentration-independent dynamics for this material at comparable pH.^26^ Interestingly, gels prepared at pH 8 showed a very subtle increase in dynamicity with concentration, as *ω*_c_ increases from 0.8 rad s^−1^ at 2 wt% to 1.1 rad s^−1^ at 5 wt%.

### Temperature-dependent strain-stiffening

**Fig. 5:**
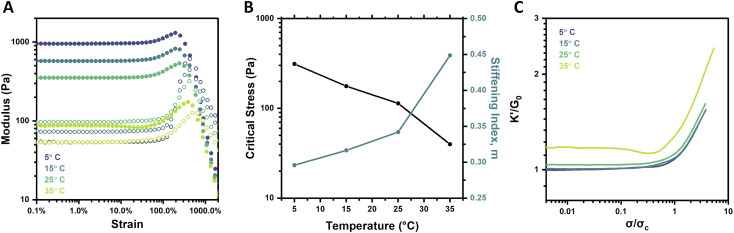
Temperature-dependent strain-stiffening behavior of 4-arm PEG gels (2.5 wt%, pH 6.5). (A) Storage modulus (*G*′, filled circles) and loss modulus (*G*′′, empty circles) as a function of strain for gels at 5–35 °C. (B) Critical stress (*σ*_c_) and stiffening index (*m*) of gels as a function of temperature. (C) Normalized differential modulus (*K*′/*G*_0_) as a function of normalized stress (*σ*/*σ*_c_) for gels at 5–35 °C.

Boronate ester crosslinks are dynamic and equilibrium-governed, and temperature thus dictates the extent of bonding and bond dynamics; such an impact is manifest in hydrogel mechanical properties. In addition to elevated temperatures serving to increase the dynamicity of the PBA–diol bonds, the mobility and flexibility of the PEG chains is also increased. Both outcomes are expected to result in weaker gels. Indeed, frequency sweeps and strain sweeps of hydrogels at 2.5 wt% and pH 6.5 over a temperature range of 5–35 °C revealed these expected trends (Fig. S10† and 5A). The highest temperature, 35 °C, resulted in the weakest and most dynamic gel, with a *G*_0_ of 87.5 Pa. The plateau modulus reliably increased to 353.4 Pa (25 °C), 575.4 Pa (15 °C), and 954.5 Pa (5 °C) as temperature was decreased. The gel tested at 35 °C also had the lowest critical stress and highest stiffening index of all four gels (Fig. 5B). The critical stress spanned approximately one order of magnitude between the 5 °C and 35 °C gels, which is a much larger range than resulted from varying pH, but smaller than that observed for varying concentration. The stiffening index spanned a range of merely 0.15 over the full temperature range assessed, in contrast to its range of approximately 0.3 for both concentration (2–5 wt%) and pH (6.5–8). This effect is also visualized when plotting *K*′/*G*_0_ as a function of *σ*/*σ*_c_, where the slopes of the stiffening regime were very similar for each temperature (Fig. 5C). Similar behavior was previously reported for synthetic, dynamic-covalent hydrogels made from branched polyethyleneimine and linear PEG.^40^ These two flexible polymers formed a hydrogel network in which stiffness was a function of temperature, but the intensity of strain-stiffening had minimal temperature-dependence. Here, the decrease in stiffness is attributed to both the increase in bond dynamicity at higher temperatures as well as a subtle shift in the binding equilibrium toward the unbound state at higher temperatures. Otherwise, the observed trend wherein the stiffening index is higher for the weaker gels formed at elevated temperature agrees with the findings for pH and concentration in this work.

### Behavior under cyclic strain

**Fig. 6:**
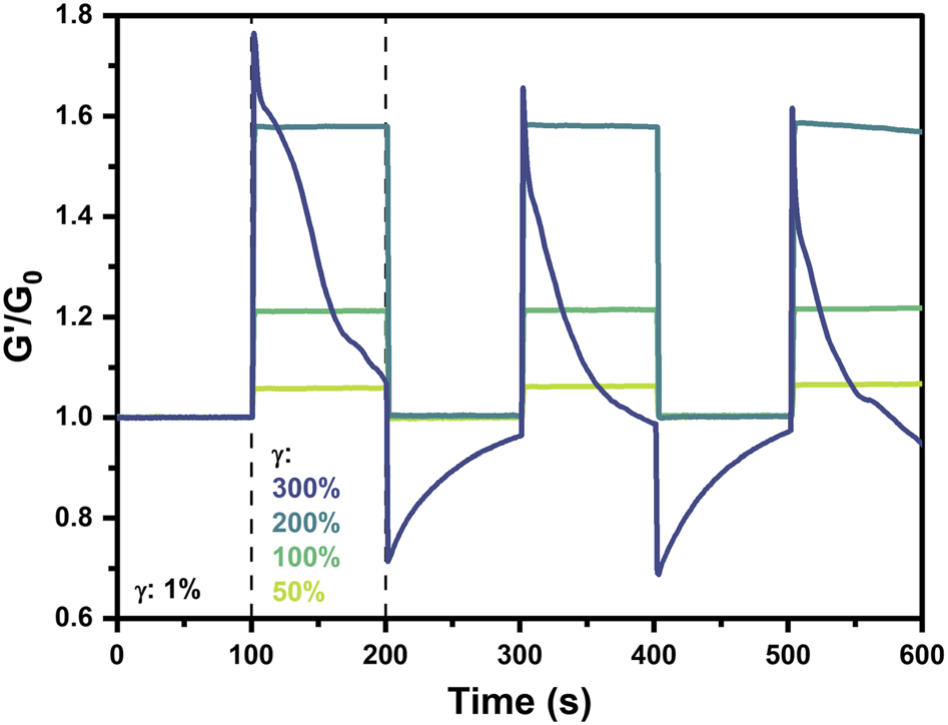
Strain-cycling behavior of 4-arm PEG hydrogels (2.5 wt%, pH 6.5, 25 °C). Storage modulus (*G*′) normalized by the initial low-strain modulus (*G*_0_) as a function of time over three cycles of low strain (1%) and high strain (50–300%) at 50 rad s^−1^.

The reversibility and repeatability of strain-stiffening in this system was next explored *via* cyclic strain measurements on 2.5 wt% gels prepared at pH 6.5. Hydrogels were alternatingly strained at 1% for 100 s followed by a higher strain (50%, 100%, 200%, or 300%) for 100 s, with this process repeated continuously for a total of three cycles. The normalized storage modulus, *G*′/*G*_0_, where *G*_0_ is the average low-strain *G*′ value over the initial period of 0–100 s at 1% strain, was presented as a function of time (Fig. 6). For strains of 50%, 100%, and 200%, each of the three cycles follows the same trend wherein gels maintain a consistent stiffness at *γ* = 1% with *G*′/*G*_0_ of 1. When strain was increased to 50%, 100%, or 200%, these hydrogels instantaneously stiffened to an extent dictated by the applied strain, reaching values of *G*′/*G*_0_ of 1.06, 1.21, and 1.58, respectively. Upon decreasing strain to 1%, the gel stiffness instantaneously reverted back to the low strain value with *G*′/*G*_0_ of 1. Accordingly, an instantaneous mechanoresponse was reliably and reproducibly demonstrated by these hydrogels, with strain-stiffening occurring upon applied strains up to 200% and recovering to the initial state following reduction in strain.

Interestingly, in the case where strain was cycled to 300%, a completely different behavior was observed. According to previously conducted strain sweeps of 2.5 wt% pH 6.5 gels at 25 °C (Fig. 2A and 3A), a strain of 300% was beyond the point where maximum stiffness was observed but before the critical yield strain where *G*′′ exceeded *G*′. In this strain cycling study, when the gel was strained to 300%, it instantaneously stiffened, similar to the gels with an applied strain of 50–200%. The immediate stiffening response in this first cycle was more pronounced than that of the 200% case, with a maximum *G*′/*G*_0_ value of 1.77. However, whereas lower values of applied strain had resulted in a plateau in the *G*′/*G*_0_ value, at 300% strain stiffness steadily decreased to a value of 1.07 by the end of the first cycle, pointing to gradual relaxation in the material. Furthermore, upon decreasing the strain to 1% to begin the second cycle, the value of *G*′/*G*_0_ instantaneously decreased to a value of 0.71, below the baseline of 1. Throughout the ensuing low-strain period, the gel appeared to recover and reached a maximum *G*′/*G*_0_ of 0.96 prior to beginning the next high-strain cycle. In this second high-strain cycle, the gel reached a maximum *G*′/*G*_0_ of 1.66, lower than that of the first cycle. The third high-strain cycle continued this trend with a maximum *G*′/*G*_0_ of 1.62. In the intervening low-strain period between the second and third high-strain cycles, stiffness instantaneously decreased and then gradually recovered to a *G*′/*G*_0_ value of 0.97. It is anticipated that longer periods of low strain beyond the 100 s explored here would result in complete recovery. On application of 300% strain, the sharp increase in stiffness followed by a steady decay to near or below the low-strain baseline speaks to an instantaneously stiffened non-equilibrium state that eventually relaxes due to self-healing-like reorganization of the dynamic-covalent bonds under high strain. The dampening of the stiffening response with each cycle also points to some type of memory for the gel strained to 300%, in which prior straining events impact its capacity for new strain-stiffening. This dampening may be due, in part, to incomplete recovery of the gel during the low strain periods. The instantaneous relaxation and subsequent recovery during the low-strain cycles following 300% strain may result from the rupture of some fraction of PBA–diol bonds under higher strain, leaving others in their bound state with their chains elastically active and supporting the network. Upon decreasing strain to 1%, these highly stiffened chains with intact bonds relax instantaneously, decreasing *G*′ to levels below *G*_0_. Because this relaxation occurs on a faster timescale than network self-healing of the dynamic bonds, *G*′ gradually recovers to *G*_0_ through network reorganization and self-healing.

### Proposed strain-stiffening mechanism

Here, a fully synthetic ideal network hydrogel composed of 4-arm PEG macromers connected *via* dynamic-covalent PBA–diol bonds was revealed to exhibit an unusual strain-stiffening response. In spite of several reports on these and other boronate ester-crosslinked 4-arm PEG networks,^26,29–31^ such strain-stiffening has yet to be observed or characterized. It has been proposed that strain-stiffening in flexible polymer-based hydrogels arises at least in part from the entropic penalty of strain-induced elongation of polymer chains, and that dynamic bonds that underlie self-healing are rarely strong enough to allow gels to reach this stiffening regime before rupturing.^41,42^ As a result, such materials typically are incapable of fully capturing both the strain-stiffening and self-healing aspects of the mechanoresponse of natural biopolymer networks. However, progress has been made recently, with certain systems containing dynamic-covalent crosslinks now capable of achieving both strain-stiffening and self-healing functionality.^41–43^ For one such work, strain-stiffening was achieved using carboxymethyl chitosan with a PEG crosslinker, where the biopolymeric chitosan affords hydrogen bonding between polymer chains and chain bundling to augment dynamic-covalent imine crosslinks in realizing strain-stiffening.^42^ In a related system, a glycosylated polyacrylamide was crosslinked with phenylboronic acid-functionalized PEG, enabling both strain-stiffening and self-healing but requiring two unique polymer chemistries to achieve this behavior. Glycosylated polyacrylamide is also likely to participate in hydrogen bonding with itself and thus is expected to exhibit some extent of bundling impacting the mechanical properties and strain-stiffening response of these gels.^41^ Another work used synthetic and biocompatible ethylene glycol-substituted polyisocyanide, which forms fibrous structures and results in hydrogels that both strain-stiffen and self-heal, yet such a system suffers from a complex design entailing heat and time to prepare the hydrogel.^43^ In all of these systems, the interactions between polymer chains contribute to their strain-stiffening response. Additionally, these systems entail either multiple polymer chemistries or relatively complex preparation to realize this effect. A recent review details similar synthetic strain-stiffening hydrogel systems, many of which are similarly complex, and offers insights into the methods used to characterize and quantify strain-stiffening responses.^44^ In contrast to these prior works on synthetic strain-stiffening systems that include dynamic-covalent bonding chemistries in combination with polymer backbones prone to bundling or entanglement, here the simple hydrogel ideal-network architecture offers advantages in preparing replicable and scalable materials. The limited ability of the PEG macromers to bundle or entangle in the dilute concentration regime studied here furthermore enables dynamic-covalent bonding to be isolated and probed for its contributions to strain-stiffening. Though the stiffening response arising from only this bonding chemistry is modest in comparison to that exhibited from (bio)polymeric systems with a propensity to bundle, these results nevertheless point to strain-responsive functionality for dynamically exchanging network bonds.

**Scheme 1:**
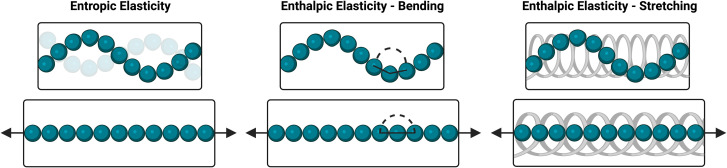
Forms of elasticity in polymer networks. Entropic elasticity (left) is a result of the loss of conformational entropy that occurs when polymer chains are straightened under strain. Enthalpic elasticity can result from bending (center) or stretching (right), in which covalent bonds in the polymer chains are unfavorably deformed under strain.

These results indicate a strain-stiffening mechanism that differs substantially from that of proteins and other biopolymers, for which both critical stress and stiffening index typically increase with increasing concentration; their stiffening index even approaches the theoretical maximum of 1.5 associated with purely entropic stiffening of semiflexible polymers.^42,45^ This stiffening behavior of biopolymers is usually attributed to a concentration-dependent increase in the density of fibrillar structures that entangle and bundle under an applied strain.^4,43,46,47^ Additionally, their nearly extended chains experience dramatic reductions in conformational entropy upon the application of relatively low strains.^45,48^ Conversely, the ideal-network hydrogels reported here lack these physical interactions that are responsible for strain-stiffening in biopolymers, with gel formation instead dependent exclusively on dynamic-covalent crosslink formation. The flexible nature of PEG also makes it likely that enthalpic elasticity will significantly contribute to the exhibited resistance to strain. Upon the application of strain, the PEG chains will be extended and aligned in the direction of strain, reducing their conformational entropy. Additionally, strain will result in unfavorable bending or stretching of covalent bonds within the PEG chains.^41,45^ Accordingly, both entropic and enthalpic elasticity are likely to contribute to the strain-stiffening of these dynamic-covalent 4aPEG gels (Scheme 1). The relationship between crosslink density and strain-stiffening is likely a result of these mechanisms, as manifest in studies here that modulate crosslink density through tuning of concentration, pH, or temperature. At lower crosslink densities, the stretching of individual polymer chains may be less restricted than in more densely crosslinked networks, allowing for a higher degree of stretching and thus a more acute stiffening response. At higher crosslink densities, chains may instead be restricted from fully stretching, reducing the magnitude of the strain-stiffening response of the gel.^41^ Accordingly, the trends observed here demonstrate a mechanistic difference from typical strain-stiffening in biopolymer networks.

Based on this proposed mechanism of stiffening resulting from polymer chain conformation, it is expected that covalent counterparts to these 4-arm PEG hydrogels would stiffen similarly. However, the strain-dependent properties of these covalent gels are rarely reported and strain-stiffening has thus not been reliably demonstrated. Two reports, including a 20 wt% gel crosslinked with thiol–maleimide bonds^49^ and a 5 wt% gel crosslinked with quasi-covalent host–guest interactions,^50^ display no strain-stiffening. It has been proposed that wall slip between the gel and the rheometer plates may prevent the visualization of strain-stiffening,^46,51^ which could impact both of these systems. Alternatively, it is possible that covalent crosslinks, similar to high polymer concentrations, restrict the extension of polymer chains and thus prevent strain-stiffening, or that dynamic-covalent crosslinks contribute to the strain-responsiveness of these materials in an unknown way. A more detailed study of ideal-network PEG hydrogels covering a spectrum of dynamic to static network bonding may be warranted to further explore this phenomenon.

An examination of these gels through the lens of large amplitude oscillatory shear (LAOS) analysis also gives insight into the impact of bond dynamics on their strain-stiffening response.^52^ Soft matter exhibits four archetypes of behavior in response to large strain. Type I is strain-thinning, in which both *G*′ and *G*′′ decrease at elevated strain.^53,54^ Type II is strain-hardening, often exhibited by biopolymers, as previously described (Fig. 1A). Type III behavior is a weak strain overshoot, in which *G*′′ exhibits a maximum, but *G*′ does not.^50,55^ Type IV behavior is characterized by maxima in both *G*′ and *G*′′, and has been attributed to molecular interactions which are weaker than those that drive strain-hardening, but stronger than those that result in weak strain overshoot. The hydrogels reported here conform to the characteristics of Type IV behavior, with maxima in *G*′ followed by maxima in *G*′′ at a slightly higher strain. The dynamic-covalent nature of the crosslinks in these gels is likely responsible for the balance of dynamics and strong intermolecular interactions, resulting in an increase in both *G*′ and *G*′′ with increasing strain. Based on the strain sweeps of these gels at different temperatures (Fig. 5A), crosslink dynamics are likely to underlie the relative increases in *G*′ and *G*′′. Crosslink dynamics increase with increases in temperature, as evidenced in frequency-dependent rheology data collected as a function of temperature (Fig. S10†). Under the most dynamically bonded state studied (35 °C), the most acute stiffening response in terms of *G*′ was observed; this gel simultaneously showed the lowest increase in *G*′′, increasing only about 73 Pa. In contrast, the less dynamic gel crosslinking afforded at 5 °C showed moderate stiffening in terms of *G*′, yet the largest increase in *G*′′ of about 559 Pa. This relationship between bond dynamics and relative increases in *G*′ and *G*′′ upon increasing strain has not been widely explored and may point to a more significant role than originally attributed to dynamic-covalent bonds in the strain-stiffening response of these materials.

## Conclusions

Strain-stiffening, although common in semiflexible biopolymer networks, has been elusive in hydrogels made from synthetic, flexible polymers and especially those prepared *via* dynamic-covalent crosslinking. This work reports strain-stiffening dynamic-covalent ideal network hydrogels made from boronate ester-crosslinked 4-arm polyethylene glycol macromers. Their strain-stiffening behavior can be altered as a function of parameters such as concentration, pH, and temperature. The inverse relationship between gel stiffness and stiffening index is universal among all three of these variables, arising due to the modulation of crosslink density by each of these factors, and supports a strain-stiffening mechanism that is unique from that of biopolymers and other semiflexible polymers. Indeed, it is proposed here that the stiffening of these flexible 4-arm PEG networks likely arises from a combination of both entropic and enthalpic elasticity. Whereas semiflexible biopolymer networks stiffen more acutely at higher concentrations—and thus higher crosslink density—the gels prepared here stiffen more dramatically at lower crosslink densities. The increased stretch on individual chains results in an increased nonlinear elastic response to strain. The strain-stiffening properties of this system, as well as its self-healing and shear-thinning abilities, biocompatibility, and simple preparation, make it a promising platform for a variety of biologically relevant engineering applications.

## Experimental methods

### Macromer synthesis

4aPEG-FPBA, 4aPEG-PyPBA, and 4aPEG-diol macromers were synthesized and characterized as previously described.^26^

### Hydrogel preparation

4aPEG-FPBA and 4aPEG-diol macromers were dissolved at 5 wt% in a pH-specific phosphate buffer (50 mM buffer salts + 100 mM NaCl) set to pH 6.5, 7, 7.5, or 8, as appropriate. To prepare 5 wt% hydrogels, these stock macromer solutions were mixed at 1 : 1 by volume. For lower concentration hydrogels (2–4 wt%), the macromer stock solutions were first diluted with the specific buffer to the target concentration prior to mixing. The resulting hydrogels were vortexed and equilibrated to ensure uniformity. The preparation of hydrogels using the 4aPEG-PyPBA macromer followed identical methods.

### Rheological characterization

Oscillatory rheology was performed on a TA Instruments Discovery HR-2 Rheometer. A 25 mm parallel plate upper geometry was used for all experiments. To minimize sample drying during testing, silicon oil was placed along the edge of the plate after sample trimming. Amplitude strain sweeps were conducted at 50 rad s^−1^ from 0.1 to 10 000% strain. Frequency sweeps were performed at 1% strain from 0.1 to 500 rad s^−1^. Cyclic strain tests were performed in order to evaluate the reversibility of the strain-stiffening response by repeatedly cycling strain between a value of 1% for 100 s to either 50%, 100%, 200%, or 300% for 100 s, repeating the cycle three times.

### Strain-stiffening analysis

Strain-stiffening was characterized by performing rheological experiments on hydrogels with a range of polymer concentrations, pH, and temperatures. The differential modulus (*K*′) is defined as the derivative of shear stress with respect to shear strain (∂*σ*/∂*γ*) in a strain sweep. When *K*′ is plotted on log–log axes as a function of shear stress, two distinct regions are present: a linear viscoelastic region of constant *K*′ at low stress, and a strain-stiffening region at high stress where *K*′ increases exponentially with stress. Critical stress (*σ*_c_) is defined as the stress where stiffening dominates the network behavior, determined from the intersection between the two linear regimes. The low-strain plateau modulus (*G*_0_) is the constant value of the storage modulus (*G*′) in the linear viscoelastic region. When *K*′ and shear stress are normalized by *G*_0_ and *σ*_c_ respectively and *K*′/*G*_0_ is plotted as a function of *σ*/*σ*_c_, similar linear viscoelastic and strain-stiffening regions are revealed. The stiffening index (*m*) offers a measure of the intensity of strain-stiffening response in the network, and is calculated as the slope of the strain-stiffening region.

## Data availability

The datasets supporting this article have been uploaded as part of the ESI.†

## Author contributions

R. C. O. and Y. X. conceived of ideas, designed/conducted experiments, and analyzed data. M. J. W. provided supervision and oversight to the work. A. M. Y. contributed to experiments and data collection. R. C. O. and M. J. W. wrote and edited the manuscript.

## Conflicts of interest

There are no conflicts to declare.

## Supplementary Material

SC-014-D3SC00011G-s001
