# Supplementary material for: Biomimetic strain-stiffening in fully synthetic dynamic-covalent hydrogel networks
Source: Chem Sci. 2023 Apr 13;14(18):4796–805. doi: 10.1039/d3sc00011g (PMC10171040; doi:10.1039/d3sc00011g)
Supplement: SC-014-D3SC00011G-s001 [file SC-014-D3SC00011G-s001.pdf]

Electronic Supplementary Material (ESI) for Chemical Science.
This journal is © The Royal Society of Chemistry 2023

Online Supporting Information

Biomimetic Strain-Stiffening in Fully Synthetic Dynamic-Covalent Hydrogel Network

Rachel C. Ollier[†], *Yuanhui Xiang*[†], *Adriana M. Yacovelli*[†], *Matthew J. Webber*[†,*]

[†]- Department of Chemical & Biomolecular Engineering, University of Notre Dame

*- Address Correspondences to: 205 McCourtney Hall, Notre Dame, IN 46556 USA; mwebber@nd.edu

Table of Contents:

I. Supporting Figures

Fig. S1- 4aPEG-PyPBA structure
Fig. S2- 4aPEG-PyPBA/4aPEG-Diol strain sweeps, 2-4 wt%
Fig. S3- Strain-stiffening properties of 4aPEG-FPBA/4aPEG-Diol gels upon drying and rehydration
Fig. S4- 4aPEG-FPBA/4aPEG-Diol frequency sweeps, pH 6.5-8
Fig. S5- 4aPEG-PyPBA/4aPEG-Diol strain sweeps, pH 6.5-7.5
Fig. S6- 4aPEG-FPBA/4aPEG-Diol frequency sweeps, pH 6.5
Fig. S7- 4aPEG-FPBA/4aPEG-Diol frequency sweeps, pH 7
Fig. S8- 4aPEG-FPBA/4aPEG-Diol frequency sweeps, pH 7.5
Fig. S9- 4aPEG-FPBA/4aPEG-Diol frequency sweeps, pH 8
Fig. S10- 4aPEG-FPBA/4aPEG-Diol frequency sweeps, 5-35°C

I. Supplemental Data:

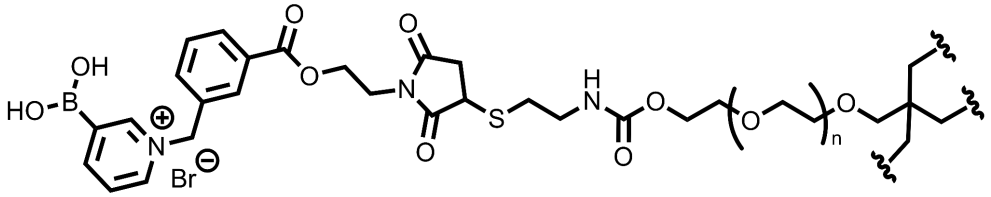

Fig. S1: Structure of 4aPEG-PyPBA.

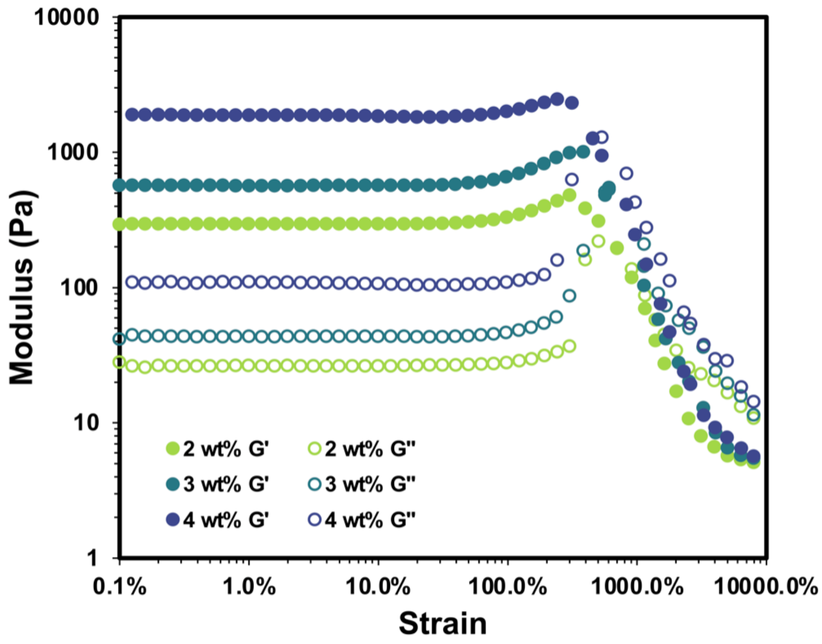

Fig. S2: Strain sweeps of 2-4 wt% 4aPEG-PyPBA/4aPEG-Diol hydrogels at 25°C, prepared at pH 7.

A

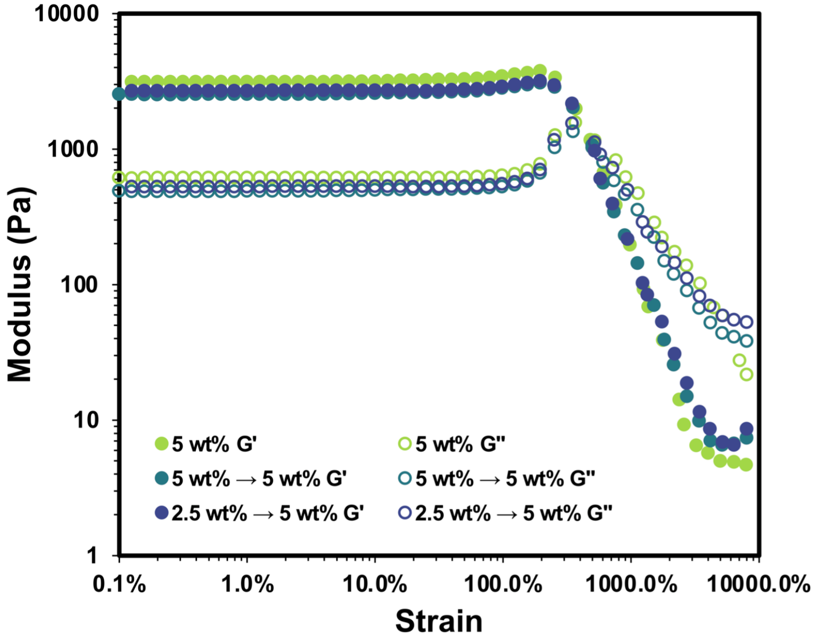

B

	Critical Stress (Pa)	Stiffening Index
5 wt%	1055.9	0.17
5 wt% → 5 wt%	1009.0	0.18
2.5 wt% → 5 wt%	1048.6	0.18

Fig. S3: Strain-stiffening behavior of 4aPEG-FPBA/4aPEG-Diol gels upon drying and rehydration. (A) Strain sweeps of a freshly prepared 5 wt% gel, a “5 wt% → 5 wt%” gel prepared at 5 wt% with 150 mM pH 6.5 buffer, lyophilized, and rehydrated to 5 wt% with DI water, and a “2.5 wt% → 5 wt%” gel prepared at 2.5 wt% with 75 mM pH 6.5 buffer, lyophilized, and rehydrated to 5 wt% with DI water. (B) Critical stress and stiffening index of these three gels.

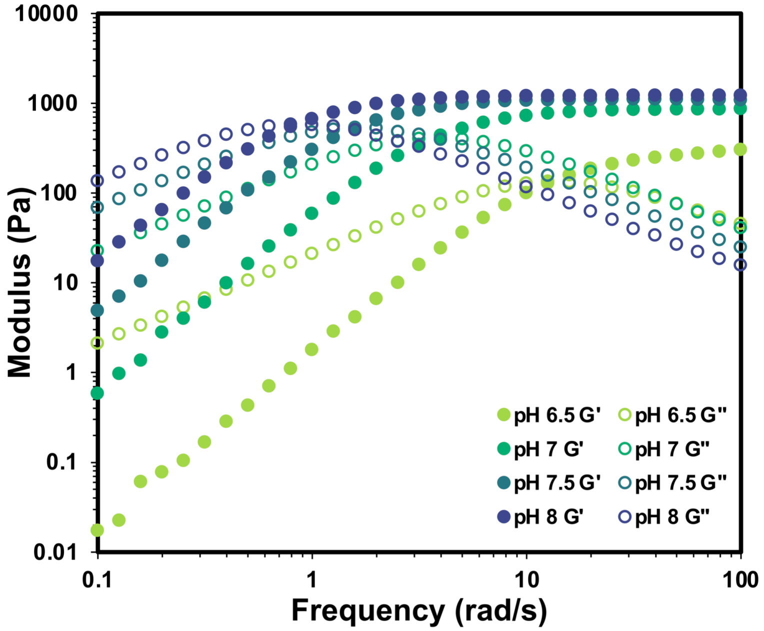

Fig. S4: Frequency sweeps of 2.5 wt% 4aPEG-FPBA/4aPEG-Diol hydrogels at 25°C, prepared at pH 6.5, 7, 7.5, and 8.

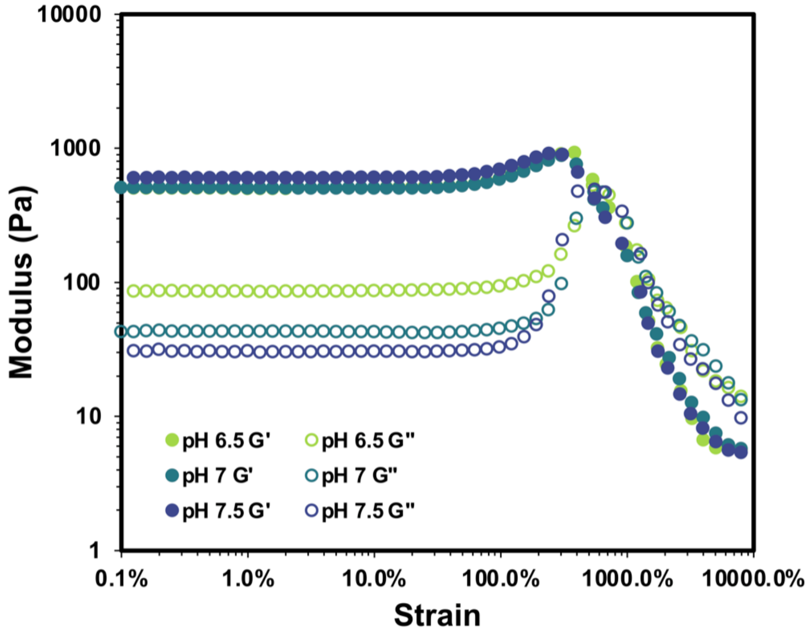

Fig. S5: Strain sweeps of 3 wt% 4aPEG-PyPBA/4aPEG-Diol hydrogels at 25°C, prepared at pH 6.5, 7, and 7.5.

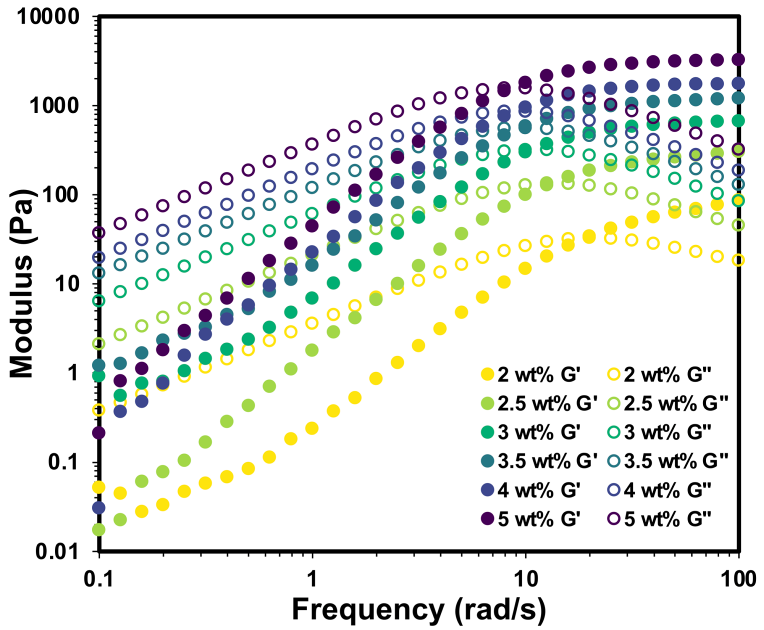

Fig. S6: Frequency sweeps of 2-5 wt% 4aPEG-FPBA/4aPEG-Diol hydrogels at 25°C, prepared at pH 6.5.

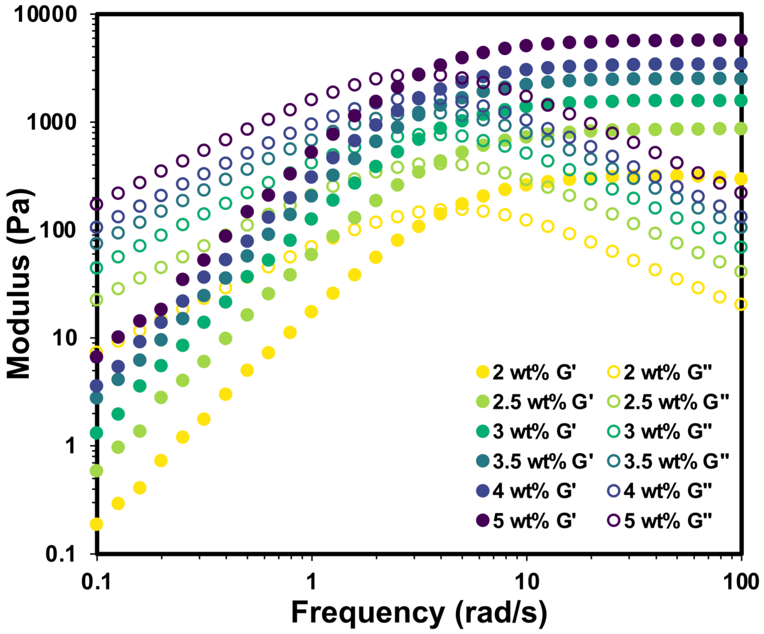

Fig. S7: Frequency sweeps of 2-5 wt% 4aPEG-FPBA/4aPEG-Diol hydrogels at 25°C, prepared at pH 7.

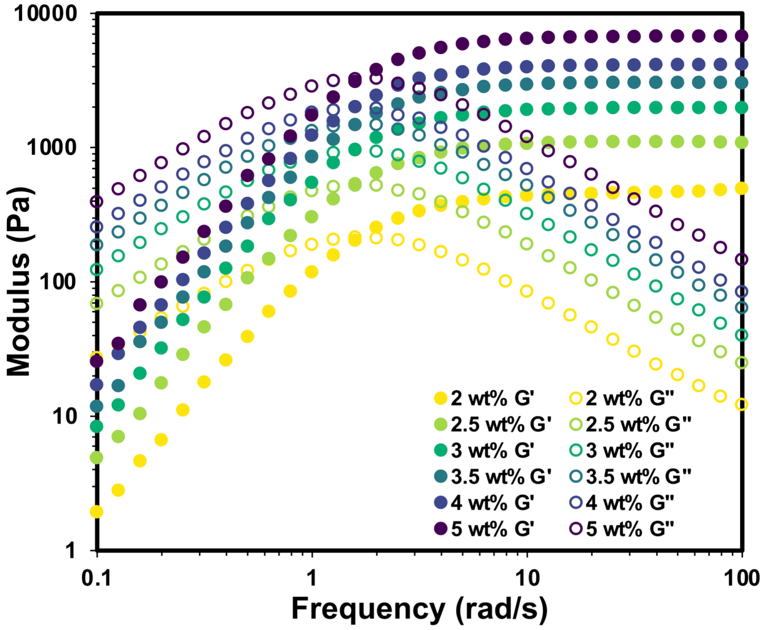

Fig. S8: Frequency sweeps of 2-5 wt% 4aPEG-FPBA/4aPEG-Diol hydrogels at 25°C, prepared at pH 7.5.

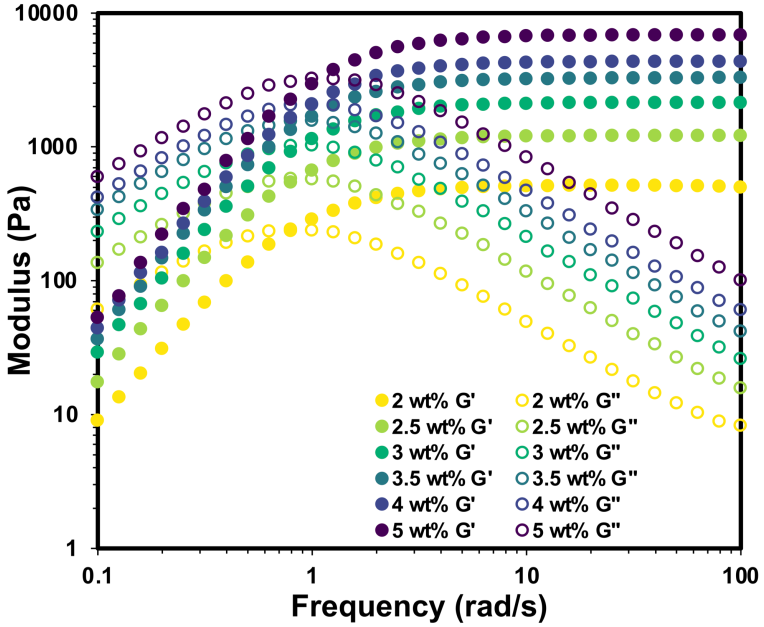

Fig. S9: Frequency sweeps of 2-5 wt% 4aPEG-FPBA/4aPEG-Diol hydrogels at 25°C, prepared at pH 8.

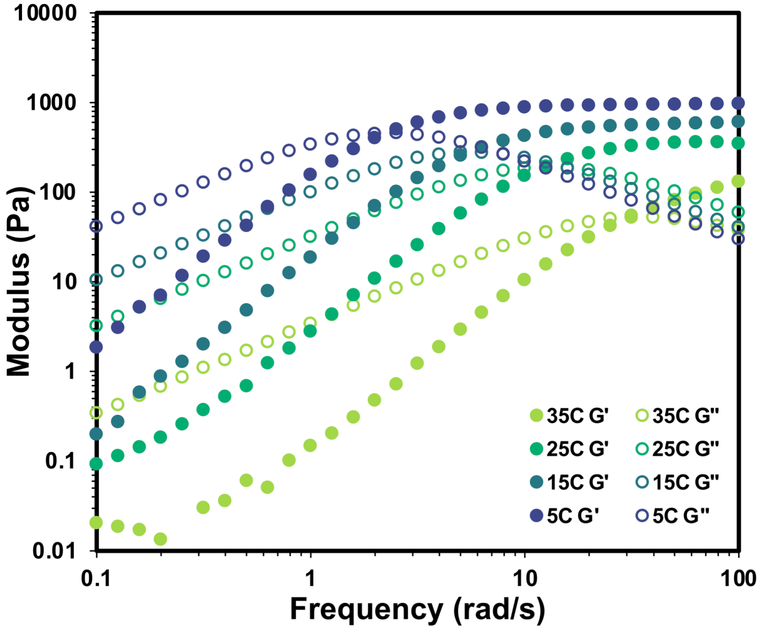

Fig. S10: Frequency sweeps of 2.5 wt% 4aPEG-FPBA/4aPEG-Diol hydrogels at 5°C, 15°C, 25°C, and 35°C, prepared at pH 6.5.
